# Resistivity of solid and liquid Fe–Ni–Si with applications to the cores of Earth, Mercury and Venus

**DOI:** 10.1038/s41598-022-14130-z

**Published:** 2022-06-15

**Authors:** Meryem Berrada, Richard A. Secco, Wenjun Yong

**Affiliations:** Department of Earth Sciences, University of Western Ontario, London, ON N6A5B7 Canada

**Keywords:** Geodynamics, Mineralogy, Inner planets, Core processes

## Abstract

Electrical resistivity measurements of Fe–10wt%Ni–10wt%Si have been performed in a multi-anvil press from 3 to 20 GPa up to 2200 K. The temperature and pressure dependences of electrical resistivity are analyzed in term of changes in the electron mean free path. Similarities in the thermal properties of Fe–Si and Fe–Ni–Si alloys suggest the effect of Ni is negligible. Electrical resistivity is used to calculate thermal conductivity via the Wiedemann–Franz law, which is then used to estimate the adiabatic heat flow. The adiabatic heat flow at the top of Earth’s core is estimated to be 14 TW from the pressure and temperature dependences of thermal conductivity in the liquid state from this study, suggesting thermal convection may still be an active source to power the dynamo depending on the estimated value taken for the heat flow through the core mantle boundary. The calculated adiabatic heat flux density of 22.7–32.1 mW/m^2^ at the top of Mercury’s core suggests a chemically driven magnetic field from 0.02 to 0.21 Gyr after formation. A thermal conductivity of 140–148 Wm^−1^ K^−1^ is estimated at the center of a Fe–10wt%Ni–10wt%Si Venusian core, suggesting the presence of a solid inner core and an outer core that is at least partially liquid.

## Introduction

The magnetic field of Mercury, Earth, and other terrestrial-type bodies is thought to originate from an internal dynamo generated by convection in an electrically conductive metallic region of the planet^1^. A dynamo may be generated when sufficient thermally and/or chemically convective motion is combined with planetary rotation to provide sufficient kinetic energy to core fluid^2^. When the heat extracted from the core through the core-mantle boundary (CMB) exceeds the heat transferred along the core adiabat, the top part of the core becomes thermally convective. When the heat extracted from the core through the CMB is less than the adiabatic heat flow, the top part of the core is thermally conductive and thermal convection is negligible. In this case, compositional convection that arises from the crystallization of the inner core (IC) becomes the dominant source of convective power^3^.The onset of chemical convection therefore indicates the upper bound for age of IC formation. In the absence of a radiogenic heat source in the core, the onset of a chemically driven dynamo depends on the release of light elements at the IC boundary (ICB) as the IC solidifies, which is governed by the light element content.

### Earth’s core composition

The composition of the metallic liquid outer core (OC) is relatively well constrained to be dominantly Fe and Ni with some light elements (e.g., O, C, S, Si and H)^4,5^. The presence of Ni is expected considering the composition of meteorites (5–10wt% Ni), while the presence of light elements is a suggested solution to the core’s density deficit^4–10^. Antonangeli et al.^11^ suggest the Si content of the Earth’s OC is 1.2–4 wt%, while core-mantle interaction models suggest up to 10.3 wt% Si^7,12,13^. In contrast, Morrison et al.^14^ reported that the density of hcp-Fe–11wt%Ni–6wt%Si (Fe_0.8_Ni_0.1_Si_0.1_) agrees with Earth’s IC density.

### Mercury’s core composition

Mercury’s present day magnetic field is approximately 1% of the intensity of Earth’s magnetic field^15^. Thermal evolution models suggest the presence of a self-sustained chemically driven dynamo as a plausible source of Mercury’s present magnetic field^16–22^. The current estimates of Mercury’s internal structure place the CMB at 1800–2100 K and 5–7 GPa and the ICB at 2200–2500 K and 36 GPa^23–26^. Models considering the presence of a thermally stratified layer of Fe–S at the top of an Fe-rich core successfully generate a dynamo of similar intensity to present-day observations^19,27–29^. This layer decreases the temperature difference across the CMB which favours a sub-adiabatic heat flux on the core side of the CMB and thus dominant chemical convection in the liquid outer core. The state of this thermally stratified layer, whether it is liquid^29,30^ or solid^31,32^, depends on its light element content. Theoretical and experimental constraints on Mercury’s core composition indicate at least 5 wt%Si^33^ and up to 25 wt%Si^34^, while 10.5 wt% Si has been recently suggested as a more plausible content^35,36^. In fact, a sub-adiabatic Fe–(7–20wt%)Si core has been shown to stimulate IC solidification and generation of a dynamo^26^.

### Venus’ core composition

Contrary to Earth and Mercury, Venus lacks a present-day magnetic field^37^. This is peculiar considering the similarity with Earth’s size and mass. The limited data on Venus’ interior are consistent with Earth’s bulk composition^38–40^. The core is thought to be electrically conductive^41^ and, although relatively weak because of a slow rotation rate, the Coriolis force provides sufficient kinetic energy to power an internal dynamo^42,43^. However, convection in the electrically conductive fluid is required to generate dynamo action^1^. The most probable interpretation of Venus’ lack of dynamo is therefore the absence of, or insufficient, convection in the core. The primordial structure of an Earth-like planet refers to a mantle composition similar to the bulk silicate Earth and a metal core that is at least partially liquid and chemically homogeneous containing 81.2wt% Fe, 7.5wt% Si, 5.1wt% Ni and other light elements such as O and S^44^. A core of solid Fe–Ni–Si (hcp-Fe–5wt%Ni–8wt%Si and hcp-Fe–5wt%Ni–4wt%Si) has recently been shown compatible with the lack of dynamo if a completely solidified core, rather than an Earth-like core, is considered^45^.

### Adiabatic heat flux

The adiabatic heat flux density on the core side of the CMB (*q*_*ad*_) is described as follows:

1 $${\mathrm{q}}_{\mathrm{ad}}=-{\mathrm{k}}_{\mathrm{c}}\frac{\mathrm{\alpha gT}}{{\mathrm{C}}_{\mathrm{p}}}$$

where *k*_*c*_ is the thermal conductivity of the core, *α* is the thermal expansion coefficient of the core fluid, *g* is the gravitational acceleration,* T* is the temperature at the top of the core, and *C*_*P*_ is the heat capacity at constant pressure (*P*) of the core fluid. Thermal conductivity of metals and metallic alloys is predominantly electron-based, while the phonon-based contribution often account for less than 2% or as much as 40% at 300 K depending on the material^46^. For example, the phonon component of thermal conductivity of Fe accounts for ~ 6.5% of the total conductivity at 1 atm and 300 K^47^, while that of Ni accounts for ~ 15% at the same conditions^46^. The electronic component of thermal conductivity (*k*_*e*_) can be calculated from measurements of electrical resistivity (*ρ*) via the Wiedemann–Franz Law (WFL):

2 $${\mathrm{k}}_{\mathrm{e}}=\frac{{\mathrm{L}}_{0}\mathrm{T}}{\rho }$$

where *L*_*0*_ is the constant theoretical Sommerfeld value (*L*_*0*_ = 2.44 × 10^–8^ WΩ K^−2^) of the Lorenz number (*L*). The value of *L* is specific to each metal and metallic-alloy and has been shown to be *P*- and *T*-dependent for Fe^48^. The Lorenz number can be determined from first-principle calculations or from independent measurements of *k* and* ρ* using multi-anvil presses or diamond-anvil cells. The effects of using the Sommerfeld value when calculating *k*_*e*_ of ternary Fe-alloys remains unclear due to the lack of literature on the Lorenz number for such systems. In this study, we investigate the thermal state of Earth, Mercury and Venus interiors using direct measurements of *ρ* of Fe–10wt%Ni–10wt%Si (hereafter referred to as Fe10Ni10Si) and use the WFL to calculate *k*_*e*_ in order to estimate *q*_*ad*_. This work is motivated by the scarce information on relevant ternary systems at the *P,T* core conditions of terrestrial-type bodies. To our knowledge, this work consists of the first multi-anvil large volume sample results in the *P* range up to 25 GPa in both solid and liquid states for a core-mimetic ternary system.

## Results

**Figure 1:**
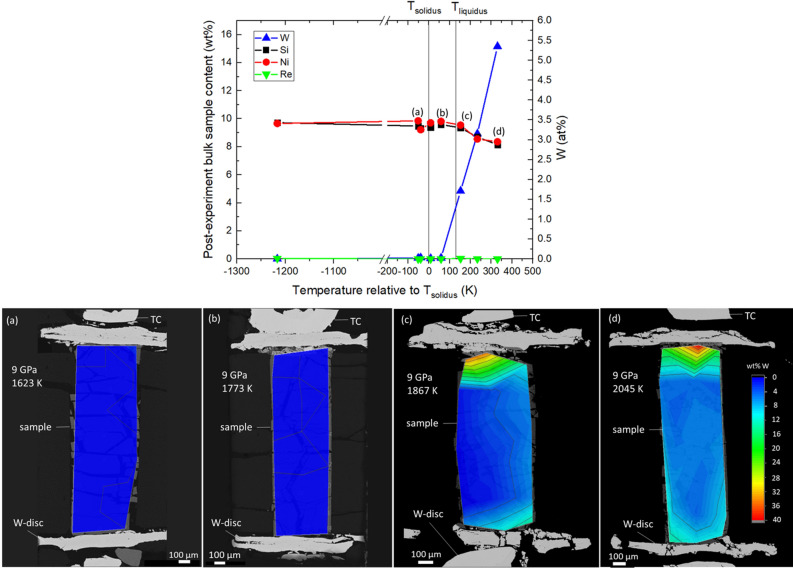
(Top) Composition of various samples from different experiments quenched from 9 GPa as a function of *T* relative to *T*_solidus_. The *T*_liquidus_ value of 1845 K at 9 GPa is displayed for reference. The left-hand side y-axis shows the abundance of all elements in wt%, while the right-hand side y-axis shows the abundance of W only in at%. (Bottom) Backscattered images from electron microprobe analysis on samples of Fe10Ni10Si quenched from 9 GPa and (**a**) 1623 K, (**b**) 1773 K, (**c**) 1867 K, and (**d**) 2045 K. The W content in each sample is illustrated using a color contour map.

Analysis of the diffusion of the W-discs into the sample at 9 GPa suggests contamination begins in the partial melting zone, and only becomes significant well into the liquid state as shown in Fig. 1. Since the focus of this study is on the *ρ* values near the liquidus *T* (*T*_*liquidus*_), contamination of the sample well above *T*_*liquidus*_ is not a concern.

**Figure 2:**
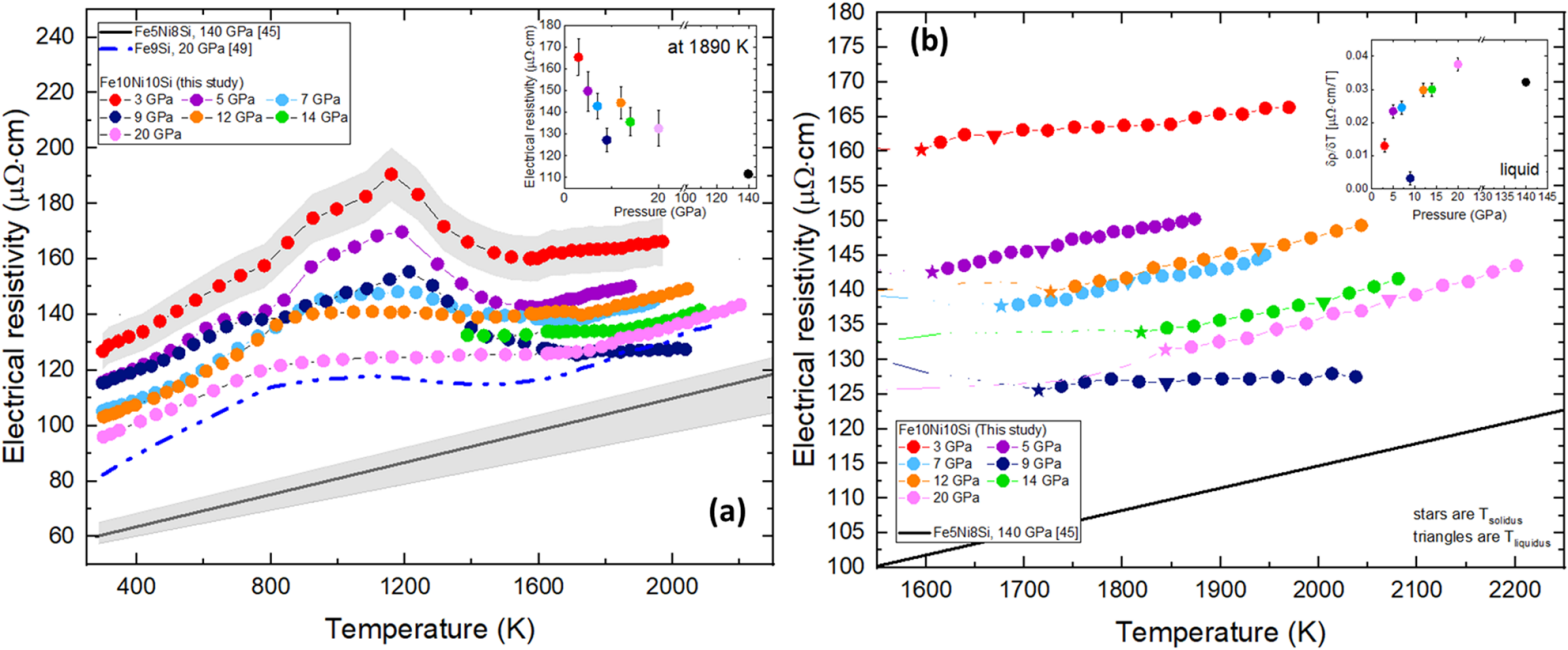
(**a**) Electrical resistivity of Fe10Ni10Si from 3 to 20 GPa as a function of *T*. The error bar in *ρ* is 4.4 µΩcm at low* T* and 8.5 µΩcm at high *T*. Results are compared to Fe8.5Si at 20 GPa^49^ and Fe5Ni8Si at 140 GPa^45^. Grey bands are shown as representative errors bars. The inset plot shows* ρ* from this study and Zhang et al.^45^ at 1890 K as a function of *P*. (**b**) Electrical resistivity in the liquid state, including the partial melting region in runs where liquid measurements are scarce. The star symbols represent *T*_*solidus*_ and the triangles represent *T*_*liquidus*_ in each data set. The inset plot shows the *ρ* slope in the liquid as a function of *P*.

The *T* dependence of* ρ* of Fe10Ni10Si is plotted in Fig. 2a. Electrical resistivity overall increases with *T* and decreases with *P*, in agreement with the recent measurements^45^ and with expectation for metallic behavior^1^. The peak observed near 1200 K, which decreases in amplitude with increasing *P*, is attributed to a possible order–disorder transition, similar to that observed in Fe8.5Si and as discussed previously^49^. Alternatively, this peak could indicate a phase transition in Fe10Ni10Si similar to the transition from bcc–fcc Fe at a similar temperature below 13 GPa although the *P*-dependence of the transition *T* is opposite to that for pure Fe. Our main results of this study do not hinge on the present qualitative interpretation of this phase since our focus is on the liquid state and the region just prior to melting. The inset of Fig. 2a shows consistency between *ρ* results at 1890 K in the partial melting and liquid state of this study and in the solid state of Zhang et al.^45^, suggesting that the effect of *P* on the isothermal *ρ* is diminished at higher pressures. This suggests there is little change in the magnitude of *ρ* on melting. This is supported by our detailed measurements and by the similar slopes of high *T* data from this study and that shown by the black line representing solid state hcp-Fe5Ni8Si^45^ in Fig. 2a,b. The beginning of the partial melting zone (e.g., 1573 K for the 3 GPa data) is defined by a slight positive change in slope near the expected melting *T*^50^. The *P*-dependence of the change in* ρ* with* T* (δ*ρ*/δ*T*) while in the partial melting region and in the liquid state seems to be negligible above 12 GPa when compared with Zhang et al.^45^ datum at 140 GPa as shown in the inset plot of Fig. 2b. At all pressures, the solidus is defined by a reversal in slope following the decreasing limb of the maximum where *ρ* begins to increase with *T*. For the liquidus, where the indication at some pressures is a slight flattening of the slope of *ρ* with *T* followed by a subtle increase, the melting boundary of Fe8Ni10Si^50^ was used as a guide.

**Figure 3:**
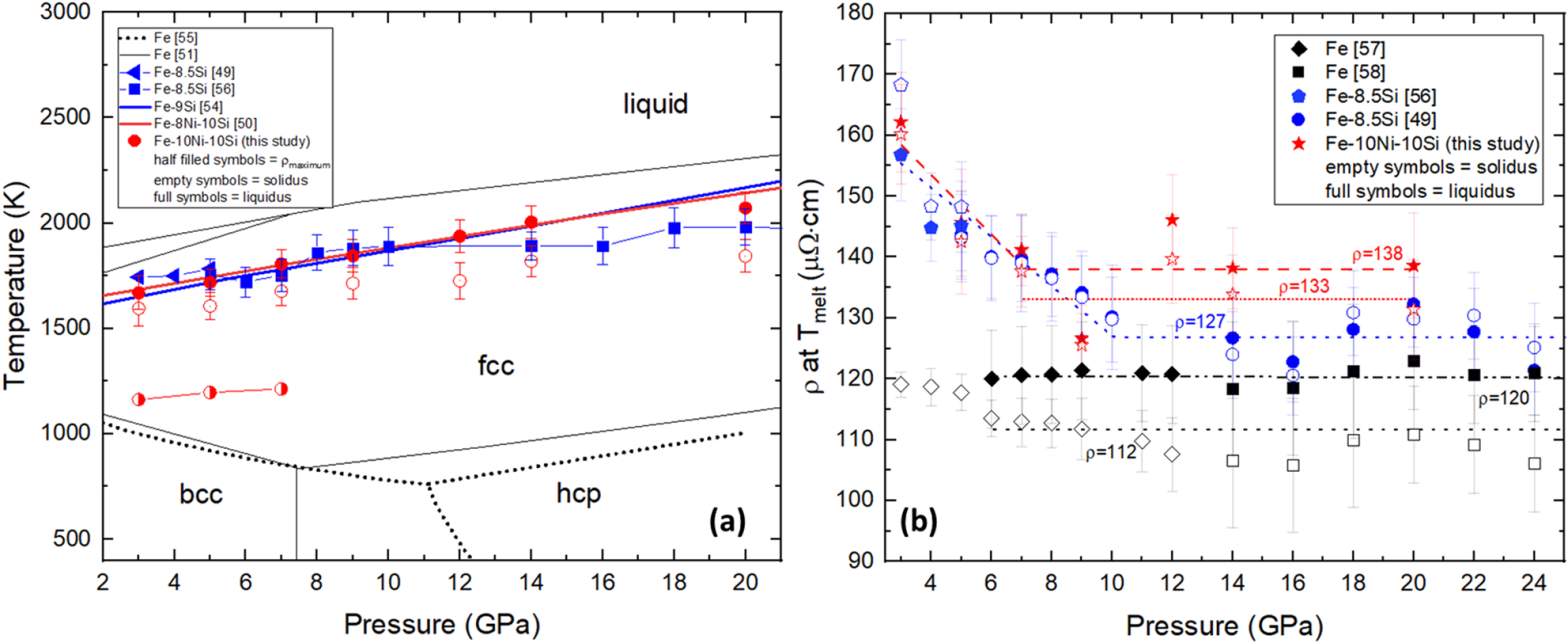
(**a**) *T*- and *P*-dependence of Fe10Ni10Si compared to the calculated phase diagram of Fe^51,55^. Comparisons are made with Fe8.5Si^49,56^, Fe9Si^54^ and Fe8Ni10Si^50^. The empty symbols represent* T* at the start of partial melting, *T*_solidus_, while filled symbols are *T* at the end of partial melting, *T*_liquidus_. The temperature at the maximum *ρ* observed near 1200 K from 3 to 7 GPa are illustrated by the half-filled symbols. (**b**) Electrical resistivity of Fe10Ni10Si near melting for various *P*. Results are compared with Fe^57,58^ and Fe8.5Si^49,56^.

Figure 3a shows the melting relations of Fe10Ni10Si. Komabayashi^51^ suggested that the melting boundary of Fe–Ni–Si alloys cannot be simply explained by the combination of Fe–Ni and Fe–Si systems. In fact, the effect of Ni on the properties of Fe alloys is expected to be small in comparison to those of light elements since Ni and Fe have similar atomic numbers^4,52,53^. Torchio et al.^52^ showed that up to 36 wt% Ni in Fe does not affect the melting curve of Fe up to 100 GPa. The similarity between the measured melting boundary of Fe8.5Si^49^ and Fe10Ni10Si can thus be explained by the negligible influence of Ni. The negligible effect of Ni can also be observed in the similarity between the melting boundaries of Fe9Si^54^ and Fe8Ni10Si^50^ extrapolated to 1 atm from their lowest *P* measurement at 20 GPa and ~ 33 GPa, respectively.

Electrical resistivity is interpreted using the mean free path of conduction electrons, which depends on their scattering rate. Scattering can be caused by (i) electron–phonon interactions (scattering by lattice vibrations), (ii) electron-magnon interactions (spin-disorder scattering), (iii) electron–electron interactions which can include *s-d* transitions in Fe, and (iv) electron-impurities interactions. At high T, scattering mechanisms (i), (ii), and (iii) become increasingly important, whereas (iv) is expected to remain independent of* T* as described by Matthiessen’s rule^59^. Deviations from Matthiessen’s rule for Fe alloys are primarily attributed to the underestimation of the spin-disorder contribution to *ρ*^60^. Deviations may also be caused by anisotropies of the Fermi surface^61^, anisotropies in electron–phonon scattering^62^, the existence of two conductivity bands (*s* and *d* bands) each having a different contribution to conductivity, or changes in the *T*-dependence of* ρ* (i.e.,* ρ* saturation)^63^. Berrada et al.^56^ suggested that the *T*-dependence of mechanism (iv) in Fe–Si alloys represents a deviation from Matthiessen’s rule. In contrast to *T*,* P* decreases the scattering rate as it suppresses the amplitude of vibration of atoms. Based on a cancellation of the* P* and* T* effects at the melting *T*, Stacey and Loper^64^ proposed that metals with a filled *d*-band (i.e., Cu, Zn, Ag and Au) would exhibit a constant* ρ* along the melting boundary. Yong et al.^58^ investigated this hypothesis with Fe and, although Fe has an unfilled *d*-band, observed a constant *ρ* behaviour from 6 to 24 GPa. Similar behaviour is observed in Fe8.5Si^49,56^ and in Fe10Ni10Si as shown in Fig. 3b. The constant* ρ* of Fe10Ni10Si is 133 µΩcm and 138 µΩ cm at *T*_solidus_ and *T*_liquidus_, respectively. These values are higher than the reported constant value of Fe8.5Si of 127 µΩcm, and that of Fe of 112 µΩ cm and 120 µΩ cm, on the solid and liquid sides of the melting boundary, respectively. The constant behaviour along the melting boundary may be attributed to insignificant changes in Fermi surface and mean free path upon melting^57^.

**Figure 4:**
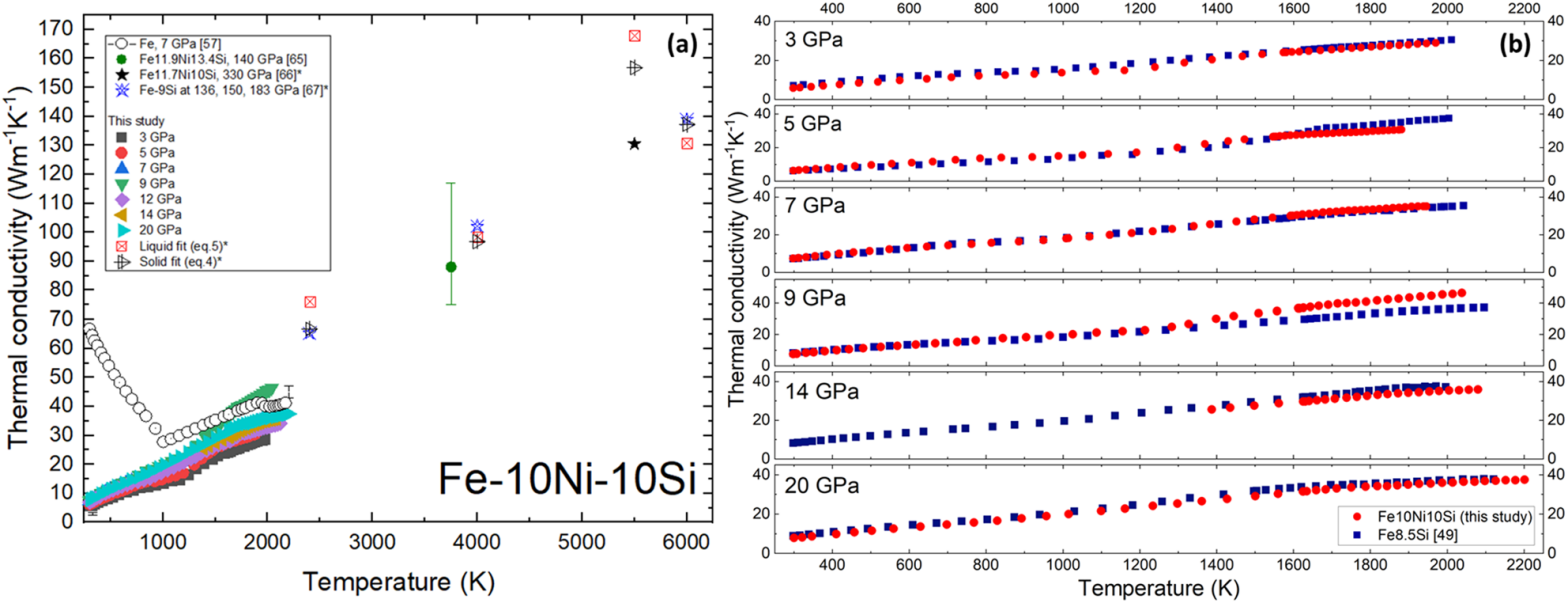
(**a**) Thermal conductivity of Fe10Ni10Si as a function of *T*. Results are compared with Fe at 7 GPa^57^, solid Fe11.9Ni13.4Si at 140 GPa^65^, solid Fe11.7Ni10Si at 330 GPa^66^, and solid Fe9Si at 136–183 GPa^67^. Equation (4) is used to calculate *k*_*e*_ of solid Fe10Ni10Si, as shown by the black triangles, at the *P*, *T* conditions of Zidane et al.^66^ and Zhang et al.^67^, shown with black and blue stars respectively. Equation (5) is used to calculate *k*_*e*_ of liquid Fe10Ni10Si, as shown by the red squares. The * denotes calculations. (**b**) Thermal conductivity of Fe10Ni10Si compared to Fe8.5Si at common* P* from 3 to 20 GPa^49^.

The calculated *k*_*e*_, shown in Fig. 4a, increases almost linearly with* T* which is different than the behaviour of pure Fe in the low temperature region but similar to the high temperature region^57^. A multi-variable linear regression analysis of the *ρ* values in the solid state from this study provides the following model of solid *k*_*e*_ (*T*, *P*):

4 $${{\mathrm{k}}_{\mathrm{e}}}_{ solid}^{3-20\mathrm{ GPa}}=-0.20 W{\mathrm{m}}^{-1}{\mathrm{K}}^{-1}+0.017 W{\mathrm{m}}^{-1}{\mathrm{K}}^{-2}*T+0.19 W{\mathrm{m}}^{-1}{\mathrm{K}}^{-1}{\mathrm{GPa}}^{-1}*\mathrm{P}$$

This relation is used to extrapolate the solid measurements to the *P*,*T* conditions of the Ohta et al.^65^, Zidane et al.^66^, and Zhang et al.^67^ solid state data, as shown in Fig. 4a. The extrapolated solid Fe10Ni10Si *k*_*e*_ are in agreement with the reported solid calculations for the nearly similar compositions of Fe9Si^67^ and Fe11.9Ni13.4Si^65^ but result in a higher value than that reported for Fe11.7Ni10Si^66^ although the compositions are similar. In addition, a multi-variable linear regression analysis of the *ρ* values in the partial melting and liquid states, see Eq. (5), from this study suggest a negligible difference between solid and liquid values at high *P*,*T*.

5 $${{\mathrm{k}}_{\mathrm{e}}}_{ liquid}^{3-20\mathrm{ GPa}}=9.3 W{\mathrm{m}}^{-1}{\mathrm{K}}^{-1}+0.011 W{\mathrm{m}}^{-1}{\mathrm{K}}^{-2}*T+0.29 W{\mathrm{m}}^{-1}{\mathrm{K}}^{-1}{\mathrm{GPa}}^{-1}*\mathrm{P}$$

Equations (4) and (5) are not founded in any elementary physical theory, they are simply linear fits of our measurements. Figure 4b illustrates the similarity in the calculated *k*_*e*_ of Fe10Ni10Si and Fe8.5Si, apart from the deviations at high* T* observed at 9 GPa. The similarities in Fe10Ni10Si and Fe8.5Si observed in the melting* T* and *k*_*e*_ suggest the addition of 10wt% Ni to Fe8.5Si has negligible effects on the properties of Fe8.5Si^49^. The similarity in the extrapolated solid and liquid Fe10Ni10Si *k*_*e*_ with the calculated Fe9Si^67^ as shown in Fig. 4a indicates the negligible effect of Ni. In contrast, the disagreement with the Fe11.7Ni10Si datum at 330 GPa^66^ indicates a steady *P*-dependence from 3 to 183 GPa, followed by an attenuated *P*-dependence up to 360 GPa. Finally, the slight variations in *k*_*e*_ of Fe10Ni10Si with* T* coincide with those observed in *ρ*, the* T* dependence of *k*_*e*_ seems to weaken at high *T*.

### Earth

Earth’s CMB (4000 K, 136 GPa) and ICB (5000 K, 330 GPa) conditions are difficult to reach in laboratory settings. Previous thermodynamic models and experimental studies considering a pure Fe core have suggested a *k*_*c*_ of 30–40 Wm^−1^ K^−1^, which indicates an adiabatic heat flow of 4–6 TW on the core side of CMB as given by Eq. (6)^64,68^.

6 $${Q}_{ad}^{core side CMB}=4\uppi {{\mathrm{r}}_{\mathrm{c}}}^{2}{\mathrm{k}}_{\mathrm{c}}{\left(\frac{\upgamma {\mathrm{g}}_{\mathrm{CMB}}\mathrm{T}}{\phi }\right)}_{\mathrm{CMB}}$$

where *r*_*c*_ is core radius (3480 km)^69^, *g*_*CMB*_ is gravitational attraction at CMB (10.68 m/s^2^)^69^, *γ* is Grüneisen’s parameter (1.5)^70^, and *ϕ* is seismic parameter (65.05 km^2^/s^2^)^69^. Zhang et al.^45^ estimated an adiabatic heat flow of 8 TW for an Fe5Ni8Si outer core from *ρ* measurements using the diamond-anvil cell. On the other hand, shock compression experiments on the melting boundary of Fe12Ni12.7Si, which has a greater Si content than Fe5Ni8Si, suggest a higher adiabatic heat flow of 13–19 TW^50^. Using Eqs. (4) and (5), we calculate *k*_*e*_ of ~ 94 W m^−1^ K^−1^ for both solid and liquid states at 4000 K and 136 GPa. Using Eq. (6) with the previous parameters, the adiabatic heat flow at the top of Earth’s core just below the CMB is estimated to be 14 TW for an Fe10Ni10Si outer core which is similar to that of Fe12Ni12.7Si^50^. The possibility of additional light elements in the core is expected to result in a lower thermal conductivity than that reported in this study, and thus a lower heat flow at the top of the core. The present-day heat flow across the CMB is not well defined and reported estimates vary considerably: 8 TW^71^, 5–15 TW^72^, ~ 15 TW^73^, ~ 5 TW^74^, ~ 12 TW^75^. Based on such a wide range of values, a reasonable conclusion on Earth’s active source of dynamo action may not yet seem possible. However, when compared to a very recent study where a multi-disciplinary and self-consistent approach was used to estimate a heat flow across the CMB of 15 TW^76^, the lower adiabatic heat flow value of 14 TW at the top of the core suggests that thermal convection may still be an active source of energy to power the geodynamo.

### Mercury

**Figure 5:**
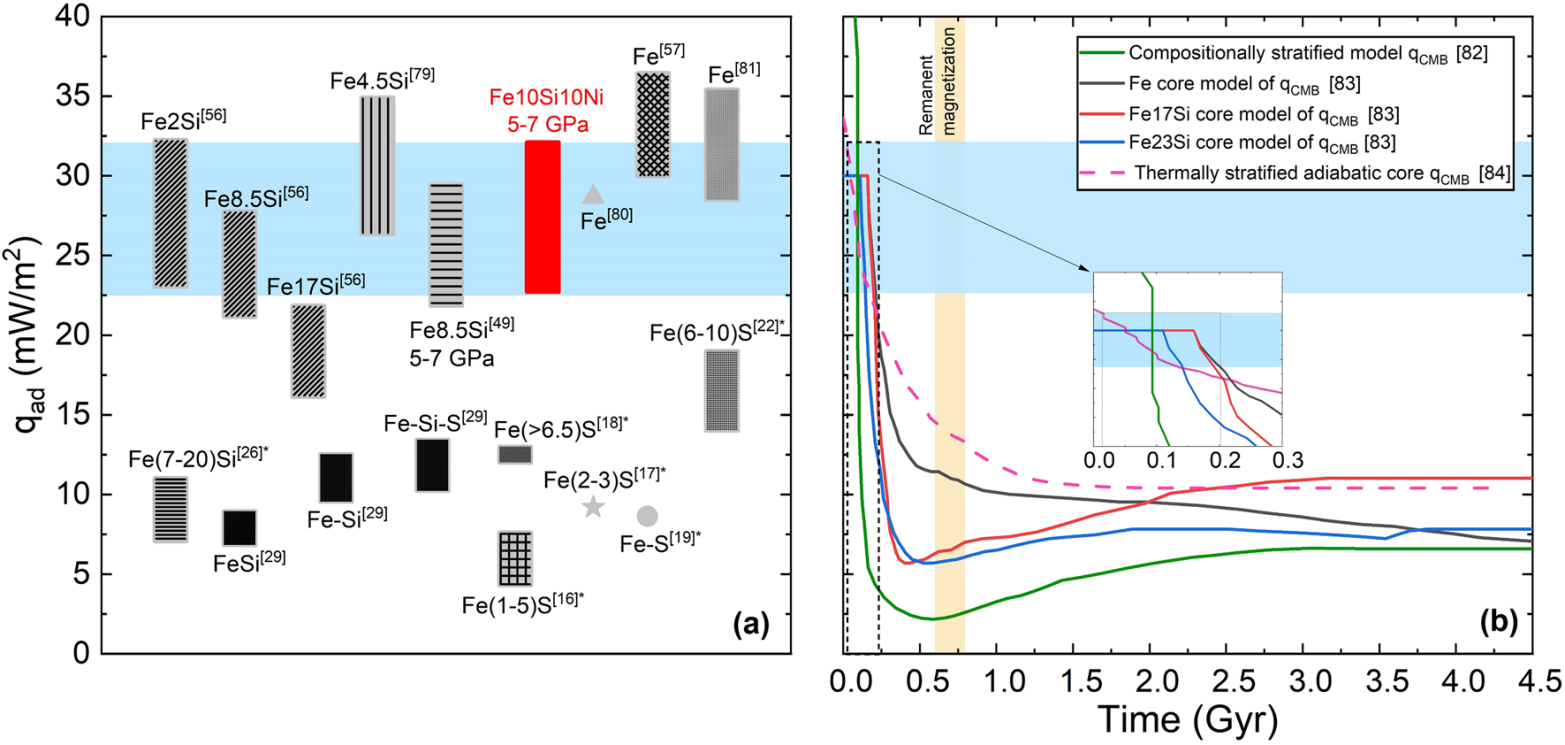
(**a**) The adiabatic heat flux density at the top of Mercury’s core compared to values calculated from other studies in the literature^16–19,22,26,29,49,56,57,79–81^. The compositions are in wt%. The results from this study are in red and extended to the blue shaded area. The * denotes theoretical studies. (**b**) Thermal evolution models of the heat flow through Mercury’s core-mantle boundary as a function of time since planet formation at 0 Gyr^82–84^. The intersection of the results from this study with the range of thermal evolution models is marked by the dotted rectangle and expanded in the inset plot. The remanent magnetization suggests the latest time interval during which a dynamo was active is represented by the orange shaded area^85–89^.

The calculated *q*_*ad*_ for Mercury using *α* of 8.9 × 10^–5^ K^−1^^48^, *g* of 4.0 m/s^2^^77^, *C*_*p*_ of 835 J Kg^−1^ K^−1^^78^, and liquid *k*_*e*_^5–7GPa^ of 29.6–35.9 Wm^−1^ K^−1^ from this study corresponds to a heat flux density of 22.7–32.1 mW/m^2^ via Eq. (1). This estimate of *q*_*ad*_ intersects models of the heat flux through the CMB (*q*_*CMB*_) between 0.02 and 0.21 Gyr after formation as shown in Fig. 5, depending on the thermal evolution model selected.

Although thermal evolution models of Fe10Ni10Si are not available in the literature, it is reasonable to assume, based on the similarities with Fe8.5Si observed in this study, that such a model would intersect the calculated *q*_*ad*_ between that of Fe and Fe17Si^83^. Within the precision of the models, this tight constraint suggests the transition from super- to sub-adiabatic heat flow through the CMB took place prior to 0.21 Gyr after formation. The thermal evolution model of Knibbe and Van Hoolst^84^, which considers a thermally stratified layer above an adiabatic core, implies a sub-adiabatic core as early as 0.02 Gyr after formation as it intersects the calculated *q*_*ad*_ from this study. As *q*_*CMB*_ becomes lower than *q*_*ad*_, at least the top of the core becomes sub-adiabatic and stimulates the nucleation of an IC. Indeed, a sub-adiabatic heat flow at the top of the core is not necessarily representative of that throughout the core as the increased* P* and latent heat with depth suggest a super-adiabatic deep core^26^. This time period is in agreement with several thermal evolution models of the core that suggest the formation of an IC, and sub-adiabatic profile, occurred within the first 0.10 Gyr^18,21,22^. Similarly, remanent magnetization observed in volcanic plains on the surface of Mercury imply an active dynamo at the time the volcanic plains formed (~ 3.7–3.9 Gyr ago) or earlier^85–89^, which is consistent with our findings.

### Venus

It has been suggested that the lack of tectonic activity on Venus produces an insulating effect that causes a high mantle* T* and therefore reduces the heat flux out of the core, resulting in the low probability of dynamo action^42,73,90^. O'Rourke et al.^91^ proposed that the lack of dynamo in Venus, assuming an Earth-like core structure, can only be explained if the core is cooling too slowly to convect, which corresponds to *k*_*c*_ of 100 Wm^−1^ K^−1^ or more. An Earth-like structure refers to a solid metallic inner core and an outer core that is at least partially liquid. Nonetheless, lower *k*_*c*_ values in the range of ∼40–50 Wm^−1^ K^−1^ favour scenarios where the lack of dynamo is due to a core that has completely solidified or preserved its primordial compositional stratification as it was unable to grow fast enough^44,73,91^. In this scenario, the mixing in the core was insufficient to generate a dynamo. Zhang et al.^45^ proposed an Fe–Ni-Si (hcp-Fe5Ni8Si and hcp-Fe5Ni4Si) core conductivity of ∼52–73 Wm^−1^ K^−1^, which promotes a sub-adiabatic core and agrees with a solid or completely stratified core scenario^91^. Using Eqs. (4) and (5), we calculate solid and liquid *k*_*e*_ of 140 Wm^−1^ K^−1^ and 148 Wm^−1^ K^−1^, respectively, at 275 GPa and 5160 K^39^. If the *P*-dependence of *ρ* is indeed attenuated above 183 GPa, these *k*_*e*_ values are overestimated although the expected values remain above 100 Wm^−1^ K^−1^. Contrary to Zhang et al.^45^, these results suggest that Venus may have a solid inner and liquid outer core^73,91^. In this case, the heat flow across the CMB does not exceed the adiabatic heat flow and thermal convection cannot take place, while suggesting that compositional convection is not enough to power Venus’ dynamo^91^. The future NASA mission VERITAS is expected to provide further constraints on the interior structure of Venus^92^.

## Discussion

The measured* ρ* of solid and liquid Fe10Ni10Si from 3 to 20 GPa shows a general increase with* T* and decrease with *P*. At melting,* ρ* remains invariant within error bars, similar to the behaviour of Fe8.5Si^49^ and Fe^58^, suggesting an invariant Fermi surface and mean free path upon melting. Further measurements are necessary to determine the extension of this behaviour at higher *P*,*T* conditions. A linear multi-variable regression analysis is done with the calculated *k*_*e*_ and the resulting relation is used to calculate the adiabatic heat flow on the core side of Earth’s CMB. A core of Fe10Ni10Si would provide a heat flow of 14 TW at the top of Earth’s core. Thermal convection appears to be an active source of energy for the geodynamo today if high values of estimates of heat flow through the CMB are used. In addition, *k*_*e*_ values between 5 and 7 GPa are used to calculate Mercury’s *q*_*ad*_. When compared to *q*_*CMB*_^82–84^, the calculated value for *q*_*ad*_ suggests a sub-adiabatic uppermost core, and thus a magnetic field primarily powered by chemical convection, between 0.02 and 0.21 Gyr after formation. Finally, *k*_*e*_ at Venus’ interior conditions is estimated and is found to be in agreement with scenarios of an Earth-like core structure, suggesting a solid inner core and at least partially liquid outer core. The conclusions are obtained assuming 10 wt% Ni and 10 wt% Si in the cores of these planets, although the exact concentration of each light element remains uncertain. When compared to the literature, our results show that the Fe–8.5wt%Si system and Fe–10wt%Ni–10wt%Si system have similar properties, suggesting that Ni could be present in a core system but not clearly observed.

## Methods

Measurements were carried out on a sample of Fe10Ni10Si at *P* from 3 to 20 GPa and at* T* up to 2200 K including the liquid state. The Fe10Ni10Si sample was initially obtained (99.95% purity, ChemPur) in powder form and then melted into a wire of 0.51 mm diameter as described by Berrada et al.^56^. The length of the sample varied according to the cell dimensions^49^. High* T* was achieved by passing an AC current (*I*) through a 0.05 mm thick cylindrical Re foil furnace surrounding the ceramic enclosed sample, while high *P* was achieved in a 3000-ton multi-anvil press. A four-wire method, as described by Silber et al.^57^, was used to pass a test current through the sample in order to obtain the voltage drop across the sample (*V*). A polarity switch reversed the current in order to expose any parasitic potentials that could be removed. Using the same four wires in an alternate mode, *T* at each end of the wire-shaped sample was also measured. A Keysight B2961 power supply provided a constant test DC of 0.2 A and a Keysight 34470A data acquisition meter operating at 20 Hz and 1 µV resolution recorded voltage. The four wires consisted of two type-C (95%W5%Re–74%W26%Re) thermocouples (TC). Contact between each end of the sample and a TC was ensured through W-discs placed between the sample and wires. The contribution of the W-discs in the voltage drop between two opposite electrodes was later subtracted using available high *P* and *T* measurements of *ρ*^93^. A combination of Ohm’s and Pouillet’s laws was used to calculate *ρ*:

3 $$\rho =\frac{\it{V}}{\it{I}}\cdot \frac{\it{A}}{\it{l}}$$

where $A$ is the cross-sectional area and $l$ the length of the sample. The uncertainties in *T* (± 5 K at low *T*, ± 25 K at high *T*), *V*, $A$ and $l$ measurements correspond to the respective standard deviations of at least 10 measurements for each data point obtained. The uncertainty on the measured geometry and averaged voltage drop at a particular *T* were used to evaluate the errors on *ρ* and *k*_*e*_. Once the target* T* was reached, *T* was quenched and the recovered sample was polished to its central cross-section in order to recover the sample geometry. The chemical composition of the sample was then analyzed with a JEOL JXA-8530F field-emission electron microprobe operating with a 50 nA probe current, a 20 kV accelerating voltage, and a 10 µm spot-size beam. Electron microprobe analysis of the starting material in wire shape indicates a homogenous sample composted of 80.48wt%Fe with 9.94wt%Ni and 9.59wt%Si. The continuity of the starting material in wire shape was confirmed with computed tomography (CT) scans with 50 µm resolution. XRD analysis performed on powder obtained from the Fe10Ni10Si wire confirmed a homogenous bcc structure of the starting sample.

## Data Availability

All experimental data are available at http://dx.doi.org/10.17632/mj37m8wcw7.1.
